# Comparison of auditory spatial bisection and minimum audible angle in front, lateral, and back space

**DOI:** 10.1038/s41598-020-62983-z

**Published:** 2020-04-14

**Authors:** Elena Aggius-Vella, Andrew J. Kolarik, Monica Gori, Silvia Cirstea, Claudio Campus, Brian C. J. Moore, Shahina Pardhan

**Affiliations:** 1 Unit for Visually Impaired People (U-VIP), Center for Human Technologies, Fondazione Istituto Italiano di Tecnologia, Genoa, Italy; 2 Dipartimento di Informatica, Bioingegneria, Robotica e Ingegneria dei Sistemi (DIBRIS) Department, University of Genoa, Genoa, Italy; 3 Vision and Eye Research Institute, School of Medicine, Anglia Ruskin University, Cambridge, United Kingdom; 4 Department of Psychology, University of Cambridge, Cambridge, United Kingdom; 5 School of Computing and Information Science, Anglia Ruskin University, Cambridge, United Kingdom

**Keywords:** Perception, Human behaviour

## Abstract

Although vision is important for calibrating auditory spatial perception, it only provides information about frontal sound sources. Previous studies of blind and sighted people support the idea that azimuthal spatial bisection in frontal space requires visual calibration, while detection of a change in azimuth (minimum audible angle, MAA) does not. The influence of vision on the ability to map frontal, lateral and back space has not been investigated. Performance in spatial bisection and MAA tasks was assessed for normally sighted blindfolded subjects using bursts of white noise presented frontally, laterally, or from the back relative to the subjects. Thresholds for both tasks were similar in frontal space, lower for the MAA task than for the bisection task in back space, and higher for the MAA task in lateral space. Two interpretations of the results are discussed, one in terms of visual calibration and the use of internal representations of source location and the other based on comparison of the magnitude or direction of change of the available binaural cues. That bisection thresholds were increased in back space relative to front space, where visual calibration information is unavailable, suggests that an internal representation of source location was used for the bisection task.

## Introduction

The human brain divides the space around the body into subspaces that are processed by different neural networks in order to generate internal representations of the external world. These representations depend on where the spatial region is relative to the body. For example, peripersonal space and extrapersonal space are defined as near the body (within reaching distance) and far from the body, respectively^1–5^. In addition, different neural mechanisms have been shown to be involved in processing left and right space^6,7^. Spatial representations can also be modified by different actions and body parts^8–11^. The ability to represent space differs depending on whether sensory feedback is available, as is the case for space in front of the individual, where vision is available, compared to back space where vision provides no information^12–16^.

One task that has been used for exploring auditory spatial representations is auditory spatial bisection, for brevity referred to hereafter as bisection. In this task the subject is presented with three successive sounds, A, B and C, and is asked to judge whether B is closer in space to A or to C. A and C are called “references” and B is called the “probe”. The bisection task involves the comparison of distances (i.e. comparing the distance between A and B, relative to the distance between B and C). It has been suggested that performance on this task requires an internal representation of source location, sometimes called an auditory spatial metric^17^, which is a mapping between physical cues such as interaural time difference (ITD) and an internal representation of space. An internal representation of source location may be built up through the processing of the “raw” spatial cues, such as ITD, interaural level difference (ILD), and pinna cues, and the spatial relations between them, usually using vision for calibration. As will be argued later, bisection does not necessarily require the use of an internal representation of source location, but nevertheless it seems plausible that such a representation would be used to perform the task.

It has been shown that in frontal space, blind subjects show larger bisection thresholds, indicating poorer performance, than sighted subjects^17–20^, perhaps because blindness limits the ability to build an internal representation of source location^17^. Bisection performance has been compared to performance for a minimum audible angle (MAA) task. In this task the subject is presented with two successive sounds, A and B, and is asked to judge whether A or B was perceived to be more to the right. A is called the “reference” and B is called the “probe”. This is a directional MAA task, distinct from a same/different MAA task. The directional component of this MAA task contrasts with the bisection task, which instead involves the comparison of distances. A directional MAA task could conceivably encourage the use of an internal representation of source location, as the task involves judgement of relative positions. However in contrast to bisection, sighted and blind subjects show similar performance for an MAA task^17–19^, because performance of this task perhaps does not depend on the use of an internal representation of source location, but rather on the discrimination of changes in the “raw” auditory cues.

Most studies of bisection have used stimuli presented in frontal space, for which vision provides precise information that could be used for construction of an internal representation of source location. To date, only one study has compared bisection in front and back space for sighted adults^12^. That study showed that, in a reverberant room, bisection was better in front space than in back space. In contrast, the MAA did not differ between front and back space. These results support the proposition that bisection depends on the use of an internal representation of source location and that prior experience with visual input is needed to build a precise spatial metric.

In the present experiment, we measured performance for azimuthal bisection and MAA tasks, for blindfolded sighted subjects, in three regions of space (front, back, and lateral relative to the subject). We chose these spatial regions because localization resolution, as measured by the MAA, is best for sounds that are straight ahead or behind, and is poorer in lateral space^21–24^. Many positions of sound sources lead to almost the same ITD and ILD, producing the so-called “cone of confusion” and requiring the use of pinna cues to distinguish front from back. ITDs and ILDs are largest for lateral locations, but they change less rapidly with azimuth than in front or back space and (near the interaural axis) they change non-monotonically with azimuth. This might increase the MAA in lateral space. In contrast, for bisection, the two reference sounds, A and C, usually have a relatively large angular separation, which would lead to larger differences between the pinna cues and only rare front-back reversals for the two reference sounds. Assuming that the bisection threshold is larger than has been measured previously for frontal sounds, at threshold the probe would be much closer to one reference than to the other, and the task could be performed by choosing the reference whose pinna cues most closely matched those of the probe. If instead an internal representation of source location is used in the bisection task, then performance should differ across the three spatial regions because of differences in the availability of visual information for calibration. Central vision is available for front space, peripheral vision is available for lateral space, and no vision is available for back space. Vision might play some role in the calibration of back space if the auditory/visual layout was viewed and then the representation was updated as the head was turned away. However, one would expect calibration in this case to be less precise than for frontal and lateral space, for which vision is constantly available.

Relative performance on MAA and bisection tasks is also affected by the environment. Tonelli *et al*.^25^ showed that in a “normal,” non-anechoic room MAA thresholds were on average lower than bisection thresholds in frontal space. However, MAA thresholds were on average higher than bisection thresholds in an anechoic room^25^, and in an echo-dampened room^19^. It is not clear why this should be the case, since the bisection task appears to be more complex, involving the assessment of three spatial positions (A, B, and C) or the comparison of two sets of spatial cues (A versus B and B versus C). To the best of our knowledge, no studies have investigated relative performance on MAA and bisection tasks in a reverberant room for different spatial regions, for which vision would be expected to play very different roles in the creation of internal representations of source location. Therefore, the second aim of the current study was to compare performance for MAA and bisection tasks in a reverberant room in the three regions of space.

In summary, the aims of the study were to: (1) Compare bisection and MAA performance for front, back, and lateral spatial regions relative to the subject, to assess the possible influence of visual calibration on the creation of internal representations of source location in different spatial regions; (2) Investigate whether and how performance for MAA and bisection tasks differs across spatial regions in a reverberant room for normally sighted adults.

## Methods

### Subjects

Eighteen normally sighted subjects (mean age: 38.5 years, SD = 8.4 years, 9 female and 9 male) were tested. All subjects confirmed that they had no cognitive impairments. Audiometric thresholds were measured using the procedure recommended by the British Society of Audiology^26^. All subjects had normal or near-normal hearing, defined as pure-tone average better-ear hearing thresholds across 0.5, 1, 2, 4, and 8 kHz ≤25 dB HL. All subjects gave written informed consent before testing commenced. The study was approved by the Anglia Ruskin Research Ethics Panel and conducted in line with the Declaration of Helsinki.

### Setup and stimuli

**Figure 1:**
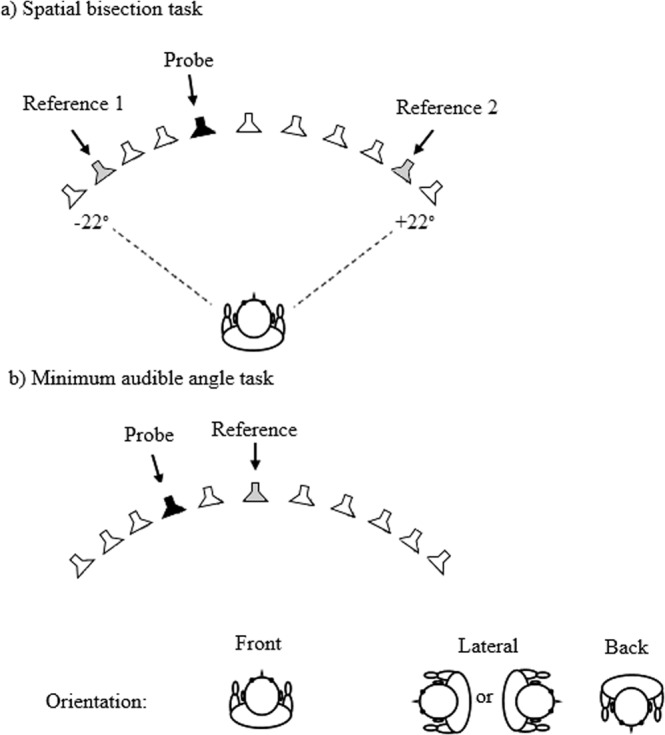
Schematic of the layout for the bisection task (**a**) and MAA task (**b**), with examples of possible reference (shown by grey loudspeakers) and probe (shown by black loudspeakers) locations. For both tasks, the subject was oriented so that sounds were presented from in front, laterally, or from the back. For illustrative purposes, all angles have been expanded relative to their true values.

Sounds were presented via an array of 11 loudspeakers, which were positioned in an arc spanning 44° on a table at a height of 1 m (Fig. 1) in a quiet room. The angular spacing of the loudspeakers was 4.4°, and the centers of the loudspeakers were separated by 10 cm. The loudspeaker array was in the approximate center of the room. The subject was seated 1.3 m from the loudspeakers, and was positioned so that the sounds were presented at 0° elevation. The room measured 7.5 (length) × 8 (width) × 3 (height) m, and had painted walls, with a tiled ceiling and carpeted floor. The room was designed to be used as a diagnostic suite for eye examinations, and tables and equipment were placed against the walls. The reverberation time (T60) was 640 ms. To avoid interference from outside noise sources, testing was performed during quiet periods when eye clinics were not run.

Subjects performed bisection and MAA tasks (Fig. 1 panels a and b, respectively), as described below. Each task was carried out for three spatial regions relative to the subject: front (with the midpoint of the loudspeaker array at 0° azimuth), lateral (midpoint of the loudspeaker array at +90° azimuth for half of the subjects, selected randomly, and −90° for the other half), or back (midpoint of the loudspeaker array at 180° azimuth).

Stimuli were white noise bursts with a frequency range from 20 to 20000 Hz, with a duration of 100 ms and 10-ms rise/fall times, sampled at 44.1 kHz with 16-bit resolution. Stimuli were presented at a level of 65 dB SPL (unweighted). The inter-stimulus interval was 500 ms. Sounds were generated using Matlab on an Asus AA185 computer with a Realtek High Definition sound card, and routed to the appropriate loudspeaker through a virtual serial port (RS485) that allowed any loudspeaker to be selected via software^27^.

### Tasks and procedures

Subjects were blindfolded before entering the test room, to prevent them having any knowledge of the room or the loudspeaker layout prior to or during testing. The position of the subject during each task was continuously monitored by the experimenter to ensure that they stayed still. Subjects were instructed that they would hear sounds originating from loudspeakers positioned around them.

#### Bisection task

For the bisection task, subjects heard three successive sounds. The positions of the first and third sounds, referred to as reference sounds, were jittered. The first reference sound was presented randomly from the loudspeaker positioned at ±22°, ±17.6°, or ±13.2° relative to the midpoint of the array, and the other reference sound was presented on the other side of the midpoint at ±13.2°, ±17.6°, or ±22°, such that the two reference sounds always had an 8-loudspeaker separation of 35.2° (e.g. the first reference was presented at −13.2° and the other reference was presented at 22°). The second sound, the probe, was presented from either the same loudspeaker as one of the reference sounds or from one of the loudspeakers between them. Subjects reported verbally whether the second stimulus was closer to the first or third sound and their response was recorded by the experimenter using a Matlab response interface. Jittering the spatial locations of the references from trial to trial prevented subjects from attending only to the position of the probe and ignoring the reference sounds.

The position of the probe for each trial was determined by the QUEST adaptive algorithm^28^, which estimated the point of subjective equality (PSE, the probe position that was perceived to be equally distant from the two reference sounds) after each response, and placed the probe for the next trial near that estimate. The position of the probe within QUEST was coded relative to the positions of the two reference sounds. Each QUEST had a span of 35.2° (8 speakers). Three QUEST runs of 20 trials each were interleaved randomly. There were 60 trials for each spatial region. Data collection lasted approximately 1 hour.

#### MAA task

For the MAA task, subjects heard two sounds in each trial. The reference sound was presented from the central loudspeaker in the array and the probe sound was presented either from the central loudspeaker or from one of the other loudspeakers. The order of the reference and probe sounds was random. For the front/back spatial regions, the task was to report whether the first or second sound was perceived to be more to the right. For the lateral region, the task was to report which sound was perceived to be located farthest forward. Responses were recorded by the experimenter using the response interface. The position of the probe was provided by a single QUEST procedure of 30 trials, which tracked the position of the probe that led to a 50% probability of it being judged to the right (or to the front) of the reference. The task took 30 minutes in total.

Performance in each spatial region (front, back, or lateral) was assessed for each task. The order of presentation of tasks and spatial region was randomized across subjects. For both tasks, no feedback was given and response time was not constrained.

##### Analysis

**Figure 2:**
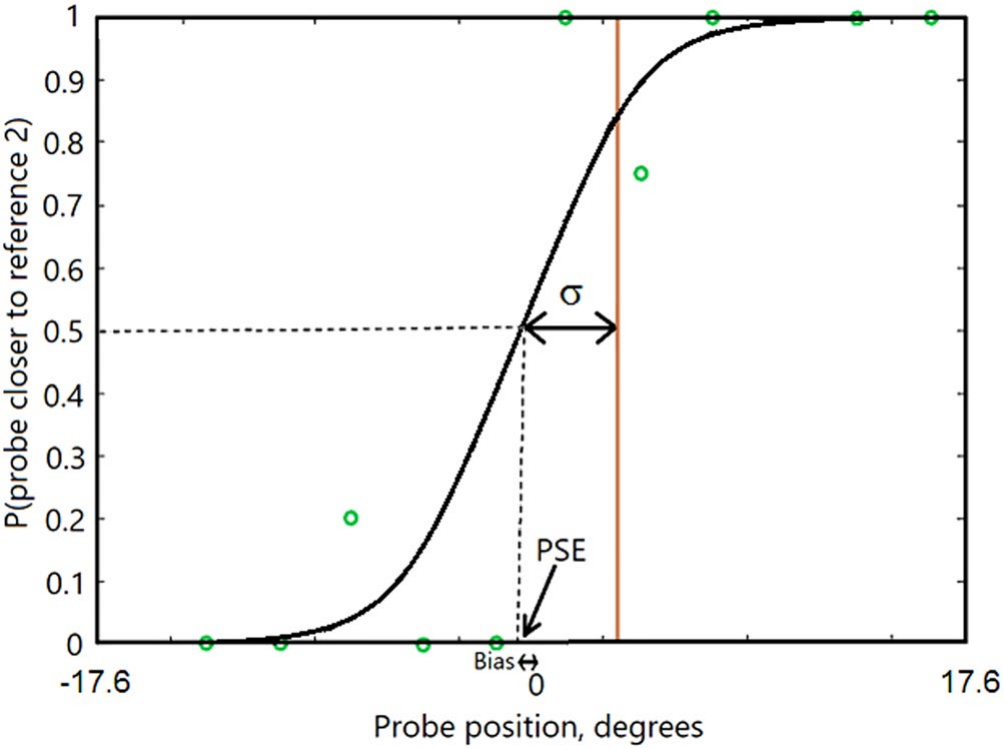
Example of a psychometric function from a typical subject for data collected in the bisection task. The standard deviation (σ) of the fit, which is proportional to the reciprocal of the slope of the psychometric function, was taken as the estimate of threshold/precision. The midpoint of the function was taken as the PSE, and the distance of the PSE from the physical center point was taken as the bias. The curve is a fitted cumulative Gaussian.

For the bisection task, the probability of the response that the second (probe) sound was closer to the rightwards (or farthest forward) reference sound was calculated for each relative position of the probe, while for the MAA task the proportion of responses ‘closer to the right position/farthest forward’ was computed for each location of the probe. Both sets of data were fitted by cumulative Gaussian functions. When fitting the psychometric function for the jittered bisection data, each probe location was first re-centered (i.e. coded in azimuth relative to the references). Figure 2 shows an example of a psychometric function for a typical subject for the bisection task. The probability of a response that the probe was closer to the rightwards reference sound is plotted as a function of relative probe position. For each subject and condition, the standard deviation (σ) of the fit, where 1/σ is proportional to the slope of the psychometric function, was taken as the estimate of threshold/precision. For the bisection task, the midpoint of the function was taken as the Point of Subjective Equality (PSE), and the distance of the PSE from the physical center point was taken as the bias. For the MAA task, the estimate of threshold/precision was again taken as the value of σ, and the mid-point of the function was taken as indicating the position at which the probe and reference appeared to be co-located. Since the order of the probe and reference sounds for the MAA task was random, any deviation of the midpoint of the function from zero indicates a bias in judging the position of the sound that varied in position (the probe) relative to the position of the sound that was fixed in position (the reference).

The data were analyzed using repeated-measures analysis of variance (ANOVA). Post hoc comparisons were conducted using paired *t*-tests for which *p* < 0.05 was considered as significant, after applying Bonferroni correction for multiple comparisons. To allow comparison of our data with previous data obtained using a fixed midpoint, in a first analysis MAA thresholds and bias were compared with bisection thresholds and bias obtained using the subset of data for reference sounds placed symmetrically around the loudspeaker at the center of the array. In a second analysis, a within-subjects ANOVA was performed on data for the three midpoints used in the bisection task, with factors midpoint, spatial region, and task.

## Results

**Figure 3:**
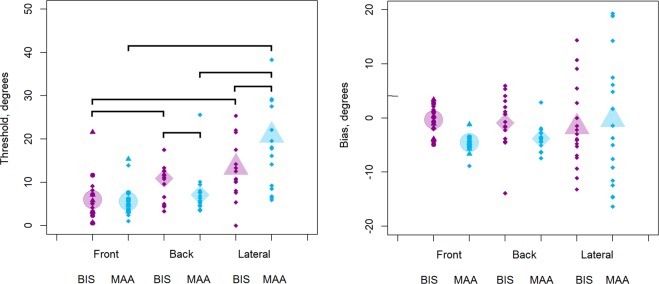
MAA and bisection (BIS) thresholds (left panel) and bias values (right panel). The thresholds/biases are shown for front (circle), back (rhombus) and lateral (triangle) positions for the bisection (BIS, violet symbols) and MAA (blue symbols) tasks. Large symbols represent means and small symbols represent data for single subjects. Brackets indicate significant differences at *p* < 0.05.

Figure 3 shows mean thresholds (left panel) and bias values (right panel) for the MAA (blue symbols) and bisection (violet symbols) tasks, for front (circles), back (rhombuses) and lateral (triangles) spatial regions. The bisection data are those obtained with the reference sounds placed symmetrically around the midpoint of the array (at ± 17.6°). A repeated-measures ANOVA was conducted on the threshold values with within-subjects factors spatial region (front, back and lateral) and task (bisection and MAA). There was a significant interaction (*F*_(2, 34)_ = 9.5, *p* < 0.01, ges = 0.12). For front space, thresholds did not differ significantly between the MAA task and the bisection task (*t*_(17)_ = −0.3, *p* = 0.7). For back space, the mean threshold was significantly lower for the MAA task than for the bisection task (*t*_(17)_ = −2.2, *p* = 0.04). For lateral space, the mean threshold was significantly higher for the MAA task than for the bisection task (*t*_(17)_ = 3.1, *p* < 0.01). A similar analysis based on bias values showed no significant effects (all *p* > 0.05). The variability of bisection and MAA bias values was higher in lateral space than in front and back space (Fig. 3).

For the bisection task (Fig. 3, violet symbols), the mean threshold was significantly lower for front space than for back space (*t*_(17)_ = −3.9, *p* < 0.01) and lateral space (*t*_(17)_ = −5.4, *p* < 0.01), consistent with the idea that auditory calibration by vision is possible and efficient only for front space. There was no significant difference between thresholds for the lateral and back spaces (*t*_(17)_ = −1.6, *p* = 0.4). As central vision is unavailable in these regions, these results are consistent with the idea that vision can be used to calibrate auditory space.

For the MAA task (Fig. 3, blue symbols), in agreement with Aggius-Vella *et al*.^12^, there was no significant difference in thresholds between front and back space (*t*_(17)_ = −1.1, *p* = 0.8), while thresholds were significantly higher for lateral (triangle) space than for both front (circle) (*t*_(17)_ = −4.8, *p* < 0.01) and back spaces (rhombus) (*t*_(17)_ = −4.4, *p* < 0.01). A similar analysis based on bias values (Fig. 3, right panel) showed no significant effects (all *p* > 0.05). Although some of the individual bias values appear quite large, especially in lateral space, this is probably simply a result of the relatively high thresholds (large values of σ), which increase the inherent variability in the estimates of bias.

In a second analysis, a repeated-measures ANOVA was performed on the thresholds and bias values for the bisection task only with the three midpoints (azimuth ± jitter) and spatial region (front, lateral, and back) as within-subjects factors. For the thresholds, there was an effect of spatial region (*F*_(2, 34)_ = 5.2, *p* = 0.01, ges = 0.07). Thresholds were significantly lower for the front than for the lateral region (*t*_(17)_ = −2.4, *p* = 0.03) and for the back than for the lateral region (*t*_(17)_ = −2.3, *p* = 0.04). There was an effect of midpoint (*F*_(2, 34)_ = 6.1, *p* < 0.01, ges = 0.03). Thresholds were significantly higher for the +jitter than for the azimuth, *t*_(17)_ = −3.2, *p* = 0.01. There was no interaction between midpoint and spatial region. A similar analysis based on the bias values showed no significant main effects or interaction. The fact that the bias did not change with midpoint indicates that subjects did indeed judge the position of the probe relative to the positions of the references, rather than judging just the position of the probe.

Although direct comparison of the current bisection data and those obtained in previous work^12,17^ is difficult because of experimental differences (data obtained in a jittered context vs. using fixed references), the bisection thresholds with the midpoint at 0° for front space were similar to the MAA thresholds, resembling previous results^12,17^ and suggesting that subjects may have used the same strategy in both jittered and fixed contexts. In contrast, for back space with the midpoint at 0°, bisection thresholds were higher than MAA thresholds. This is consistent with the idea that the bisection task was performed by making use of an internal representation of source location, and that lack of vision for back space led to a less precise internal representation^12^. Our data extend previous findings regarding auditory spatial perception^8,12,14–16,26,29^, by showing different relative performance on bisection and MAA tasks for front, back and lateral spatial regions.

## Discussion

We measured performance for bisection and MAA tasks in different regions of space in a reverberant environment. For front space thresholds for the two tasks were similar, for back space thresholds were lower for the MAA task than for the bisection task, and for lateral space MAA thresholds were higher than bisection thresholds and also higher than MAA thresholds obtained in the front and back spaces. In what follows, we first compare our results to previous related results. We then consider two possible explanations for the pattern of the results, one based on the use of internal spatial representations to perform the bisection task and one based on the use of the “raw” acoustic cues.

In the current study, MAA thresholds in lateral space were approximately 4 and 3 times as large as MAAs in front and back space, respectively, similar to the pattern of results reported by Saberi *et al*., who found lateral MAAs to be approximately 5.5 times as large as MAAs in front and back space. We found that MAA thresholds did not differ significantly for front and back space, consistent with the results of Aggius-Vella *et al*.^12^. For front space, we found similar bisection and MAA thresholds in a reverberant room, whereas Tonelli *et al*.^25^ reported that in a reverberant room MAA thresholds were on average lower than bisection thresholds. Although Tonelli *et al*.^25^ did not report the reverberation time of their “normal” room, making comparisons difficult, the difference between the current findings and those of Tonelli *et al*.^25^ may be due to differences in the acoustic characteristics of the testing rooms. Room reverberation has been shown to reduce accuracy for localizing broadband sounds in azimuth^30^ and it is possible that room reflections had a greater impact on performance for the bisection task in the study of Tonelli *et al*.^25^ because that task involves comparison of the internal representations of position or interaural cues of three sounds, while the MAA task involves only two sounds. Our findings and those of Tonelli *et al*.^25^ in their “normal” reverberant room contrast with those observed in anechoic or echo-dampened environments, for which MAA thresholds have been reported to be on average higher than bisection thresholds^19,25^.

It should be noted that we jittered the positions of the reference sounds in the bisection task while the previous experiments discussed above did not use jitter. The use of jitter may have made the task slightly harder. We did not use jitter in the MAA task, and this may have affected relative performance for the bisection and MAA tasks. However, we would not expect this to affect the pattern of results across different spatial regions.

We now discuss two possible explanations of the pattern of results, the first in terms of the use of internal spatial representations and the second in terms of the use of “raw” physical auditory cues.

### The use of internal spatial representations

The results for the front and back spaces are in line with previous findings showing that bisection performance is better for front space than for back space^12,17^. This is consistent with the idea that the bisection task involves the use of internal spatial representations and that the availability of visual information for calibration determines the precision of those representations. Further supporting this idea, blind subjects show poorer bisection than sighted subjects in front space^17–20^ (to date, no studies have investigated bisection performance for blind subjects in back space). In contrast, blind and sighted subjects perform similarly for an MAA task^19^, probably because this task does not require the use of an internal spatial representation, whereas the bisection task may usually depend on such a representation.

For lateral space, our results showed that MAA thresholds were significantly higher than bisection thresholds. This may have occurred because front-back ambiguities and localization blur (i.e. uncertainty of mapping to a spatial representation) have a strong deleterious effect on MAA thresholds, while they may play a smaller role for the bisection task. In our study the two reference sounds, A and C, had a relatively large angular separation of ±17.6° from the midpoint. This is larger than the MAA for lateral space, making it likely that robust pinna cues were available for the two reference sounds and leading to few front-back reversals for those sounds.

Both bisection and MAA thresholds were significantly higher for lateral space than for frontal space. This is probably partly a result of the fact that ITD and ILD cues change only slightly, and non-monotonically, with sound azimuth in lateral space, while they change much more in frontal space. It seems likely that pinna cues played a dominant role in lateral space for both tasks, and these cues afford less precise localization than ITD and ILD cues do in the front and rear where those cues vary maximally and monotonically with azimuth.

Brimijoin^31^ recently reported that for moving sound sources auditory space is dilated about the midline in front space and compressed about the interaural axis, such that a sound source must move twice as far when laterally located to have the same perceived displacement as a sound moving across the midline. Similar effects were predicted for static sources. If bisection involves the comparison of internal representations of sound position, Brimijoin’s results suggest an additional factor that may contribute to our finding that the mean bisection threshold for lateral space was approximately twice as large as that for frontal space. A perceptual expansion of frontal space has also been reported for visual space and is referred to as the cortical magnification factor^32^. The expansion of perceptual space for frontal targets relative to lateral targets (where vision is less precise) for both vision and audition may reflect the role that visual information plays in calibrating auditory space^17^. In the current study, bisection thresholds in back space, where vision is unavailable, were not significantly different from lateral thresholds, consistent with this viewpoint.

### Comparison of the magnitude or direction of change of the binaural cues to perform the MAA and bisection tasks

Subjects might use a strategy of comparing the magnitude or direction of change of the binaural cues, without necessarily using an internal representation of source location, and changes in the raw pinna cues might also be used. In the MAA task, the subject could compare the ITD/ILD of the two stimuli. For example, for the front region, the stimulus with the more positive (or less negative) ITD/ILD would be judged as more to the right. This strategy would work well for front and back space, but would not be effective for lateral space, for which a decrease in ITD/ILD could indicate a sound that was more towards the front or more towards the back. Some other cue, such as changes in spectrum produced by the pinnae, must be used to perform the MAA task in lateral space. That the ITD/ILD comparison strategy is less effective when applied in the lateral region of space than for the front and back regions could explain why MAA thresholds were significantly and markedly higher for lateral space than for front or back space.

**Figure 4:**
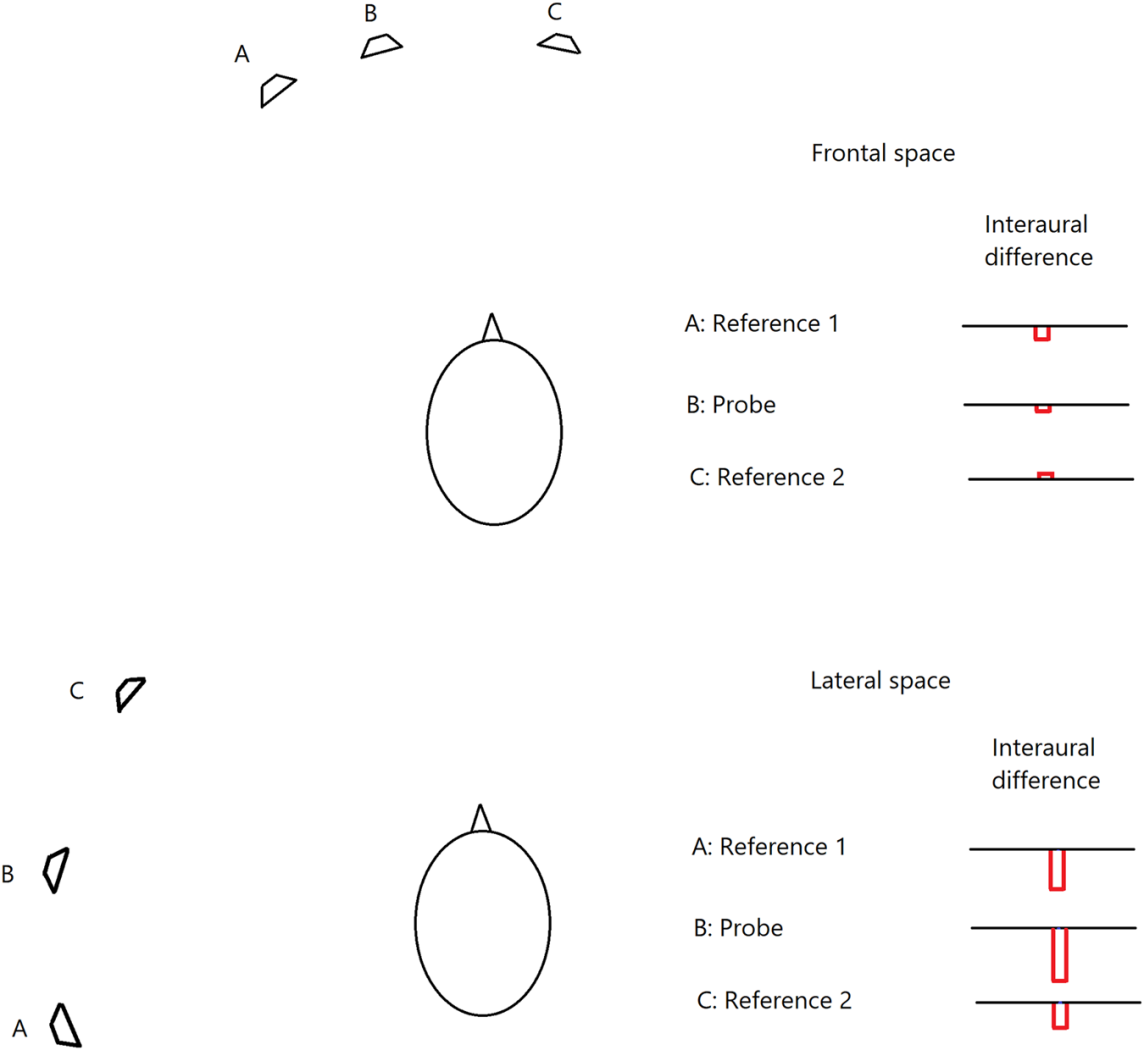
Illustration of how subjects could compare the magnitudes of binaural cues to perform the bisection task for frontal space (top) and lateral space (bottom). The lengths of the red open bars show schematically the magnitudes of the interaural cues. Cues favoring the left ear are plotted below the horizontal lines and cues favoring the right ear are plotted above the lines.

Consider now the bisection task. This could be performed by picking the reference stimulus whose ITD and ILD most closely matched those of the probe, as illustrated in Fig. 4. In that figure, an interaural difference favoring the left ear (e.g. a higher level or a lead in time at the left ear) is represented by a red bar below the horizontal line, and an interaural difference favoring the right ear is represented by a red bar above the line. In the example for frontal space (top), the midpoint of the two reference sounds is slightly to the left. The interaural difference favors the left ear for reference A and the probe and favors the right ear for reference C. The interaural difference for the probe is closer to that for reference A than it is to that for reference C leading to a response that the probe is closer to reference A. In the example for lateral space (bottom), the midpoint of the two reference sounds is slightly to the front. The interaural difference strongly favors the left ear for reference A and the probe and favors the left ear more weakly for reference C. The interaural difference for the probe is closer to that for reference A than it is to that for reference C, leading to a response that the probe was closer to reference A. A problem with this account for lateral space is that front-back ambiguities would compromise the use of ITD and ILD cues, unless the ambiguities were resolved by the use of pinna cues, as described earlier.

The fact that bisection thresholds were higher for the lateral region than for front or back regions could be partly explained by the relatively small physical changes in ITD/ILD with changes in azimuth in the lateral region, which would make it harder to compare the magnitudes of differences in ITD/ILD and would lead to a reliance on pinna cues. At the average bisection threshold, about 13.5°, the probe was almost coincident with one reference and considerably separated from the other (given that the two reference sounds were separated by 35.2°), so the task could be performed by choosing the reference whose pinna cues most closely matched those of the probe.

The finding that MAA thresholds were higher than bisection thresholds in lateral space can be explained by the available cues. As explained earlier, in the bisection task for lateral space subjects probably relied strongly on pinna cues; at the mean measured threshold, the pinna cues for the probe would resemble the pinna cues for the nearer reference much more closely than they would match the pinna cues for the farther reference. In contrast, at the MAA threshold in lateral space (about 22°), the pinna cues for the reference and probe would differ by a relatively small amount.

There is, however, a difficulty with an explanation based simply on the use of “raw” cues, without the use of spatial metrics. Such an explanation leads to the prediction that relative performance on the MAA and bisection tasks should be the same for front and back space. In fact, performance for front space was very similar for the MAA and bisection tasks, while performance for back space was worse for the bisection task than for the MAA task. Another difficulty with an explanation based on the use of “raw” cues is that blind subjects perform more poorly for the bisection task than for the MAA task in front space^17–19^, while our results and those of others for sighted subjects show very similar performance for the two tasks^33–35^. The effect of blindness is consistent with the idea that performance of the bisection task depends on the use of an internal representation of source location and that this representation is less well calibrated for blind subjects.

## Conclusions

Bisection and MAA thresholds were measured for normally sighted subjects for sound sources presented in three spatial regions in a reverberant room. Thresholds for the two tasks were similar in frontal space. In back space thresholds were lower for the MAA task than for the bisection task, while for lateral space the pattern was reversed, a finding that to the best of our knowledge is novel. While some aspects of the results can be explained in terms of the use of “raw” auditory cues, the overall pattern of the results supports the idea that performance of the bisection task usually depends on the use of an auditory internal representation of space and that this representation is best calibrated for front space, where visual information is available.
